# Long-term effects on depression and anxiety of an internet-based stepped care intervention for patients with cancer and symptoms of depression and anxiety. The U-CARE AdultCan trial

**DOI:** 10.1016/j.invent.2023.100625

**Published:** 2023-05-02

**Authors:** Helena Igelström, Maria Carlsson, Anna Hauffman, Louise von Essen, Helena Grönqvist, Birgitta Johansson, Erik M.G. Olsson

**Affiliations:** a Department of Women's and Children's Health, Uppsala University, Akademiska sjukhuset, 751 85 Uppsala, Sweden; b Department of Public Health and Caring Sciences, Uppsala University, Box 564, 751 22 Uppsala, Sweden; c Department of Surgical Sciences, Uppsala University, Akademiska sjukhuset, Entrance 78, 1st floor, 751 85 Uppsala, Sweden; d Department of Immunology, Genetics, and Pathology, Uppsala University, Rudbecklaboratoriet, 75185 Uppsala, Sweden

**Keywords:** Anxiety, Cancer, Depression, Long-term follow-up, Psycho-educational intervention, Randomized controlled trial

## Abstract

**Background:**

Cancer is a serious disease that commonly causes significant psychological distress. The internet-based intervention (iCAN-DO), utilizing a stepped care approach for the treatment of anxiety and depression in individuals with cancer, has been shown to have favorable results for symptoms of depression at the primary endpoint, 10 months after randomization compared to standard care (SC). The aim of the present study was to evaluate the long-term effects of the intervention 18 and 24 months after randomization.

**Methods:**

Patients with breast, colorectal, or prostate cancer and a score > 7 on either of the Hospital Anxiety and Depression Scale (HADS) subscales (n = 245) were recruited to the study in conjunction with a regular hospital visit. They were randomized to access to the stepwise iCAN-DO intervention for 24 months or to SC. Step 1 of the intervention comprised psycho-educative online material. In Step 2, internet-based cognitive-behavioral therapy with individual online support from a therapist was added. Step 2 was offered to those who reported persistent anxiety or depression symptoms (>7 on HADS), also at 1, 4, and/or 7 months after randomization. Missing data were imputed using the last rank carried forward method and used for the main analyses according to the intention-to-treat approach. Effects sizes (Cohen's *d*), and minimally clinically important difference (MCID) were calculated. Linear mixed models were used to analyze intervention effects over time.

**Results:**

Symptoms of depression decreased significantly (*p* < 0.05) in the iCAN-DO group compared with the SC group from baseline to 18 months (*d* = 0.29), but not to 24 months (*d* = 0.27). Even though the average iCAN-DO group participant surpassed a MCID in symptoms of anxiety (>2 p) at both long-term follow-ups, the differences did not reach statistical significance, either at 18 months (*p* = 0.10) or 24 months (*p* = 0.09). Positive effects of iCAN-DO compared with the SC were also shown for some secondary HRQoL-outcomes; social functioning at 18 months (*p* = 0.02) and 24 months (*p* = 0.001), and sleep problems at 24 months (*p* = 0.01).

**Conclusion:**

A stepped-care internet-based intervention that has previously shown positive results for symptoms of depression at 10 months did show similar positive long-term effects also at 18 months. For symptoms of anxiety, no effect could be shown. The internet may provide an effective format for interventions to reduce symptoms of depression after cancer at patients' own choice of time, regardless of distance to a psycho-oncology clinic.

## Highlights

• A cancer diagnosis may cause significant psychological suffering.
• Internet-delivered support offers care flexible in time and place.
• The intervention iCAN-DO reduced depression symptoms 18 months after randomization.
• The results are limited to a quite healthy sub-population of persons with cancer.

## 1 Introduction

Facing a diagnosis of cancer often entails a shock, which may give rise to uncertainty and worry. The cancer treatments are commonly lengthy, usually complex, and often cause side-effects that can be long-lasting. Even after treatment, persons treated for cancer can continue to face a variety of physical, mental, social, or existential problems. However, the prevalence of symptoms of anxiety and/or depression during and after cancer treatment varies in studies (10–60 %) depending on diagnosis and time point (Lopes et al., 2022; Maass et al., 2015; Mitchell et al., 2011; Thalén-Lindström et al., 2017). Psychological interventions have been evaluated but have often included unscreened participants or participants with a low level of distress at baseline and thus with low effectiveness due to little room for improvement (Sanjida et al., 2018). Therefore, it is important to perform screening in order to identify individuals who need extra psychosocial support when evaluating a psychological intervention.

Psycho-educational support can reduce distress in individuals treated for cancer (Galway et al., 2012). Such support encompasses activities that combine education and other activities, such as counseling and supportive interventions. Specifically, it means providing individuals with information about treatments, symptoms, resources and services, training to respond to disease-related problems, and teaching problem-solving strategies to enable patients to cope with the illness and to improve the adherence to cancer treatment. A systematic review and meta-analysis about effects of internet-based psycho-educational interventions among individuals with cancer (Wang et al., 2020) showed effects on symptoms of depression and fatigue, but there was no evidence for effects on quality of life or distress. The only study that specifically measured anxiety showed a significant effect. However, the meta-analysis only included seven studies so the evidence is so far quite restricted and especially regarding the long-term effects, with the longest follow-up time being 12-months and only in one study (Wang et al., 2020).

Cognitive-behavioral therapy (CBT) can be a useful technique to understand thoughts, feelings and behaviors that can cause or maintain symptoms of anxiety or depression in patients with cancer (Pitman et al., 2018). Internet-based cognitive-behavioral therapy (iCBT) is gaining more attention at the present time due to its accessibility. If the psycho-educational support is not enough to relieve distress, psychological treatment e.g., iCBT, could be added as a second step in a stepped care approach. Such an approach means that interventions with different intensities are provided at different times. Treatment effects are repeatedly evaluated, and individuals who do not respond to one level of support are transferred to the next level, where they receive more intensive support (Bower and Gilbody, 2005). Stepped care has successfully been used for treatment of symptoms of anxiety and depression (Krebber et al., 2016; Fann et al., 2009) and for stress management among individuals with cancer (Arving et al., 2019).

Among individuals with cancer, the use of the internet to provide support has increased over the years and is considered by many as a significant source of support (Tarver et al., 2018). The search for information is the most common activity, but visiting online peer support networks, blogs, and social networks is also common (Beckjord et al., 2008; Mattsson et al., 2017), and has been found to be valuable. Individuals can seek treatment from home or other locations at their convenience, which is an important aspect for individuals fatigued by the disease and treatment (Leykin et al., 2012). For some people, seeking psychological therapies is connected with a stigma; thus, privacy and confidentiality are additional benefits of providing interventions online.

The AdultCan study is part of the strategic research program U-CARE (The Uppsala University Psychosocial Care Program) supported by the Swedish government. Within U-CARE, an internet-based infrastructure (the U-CARE-portal) has been developed for delivering and evaluating internet-based interventions. The main result in the AdultCan study (at the 10-month follow-up) was that symptoms of depression decreased significantly in individuals randomized to the iCAN-DO intervention (described below) compared to standard care (SC), as opposed to symptoms of anxiety (Hauffman et al., 2020a).

The aim of this study was to evaluate the long-term effects (18 and 24 months after randomization) of the stepped-care internet-based intervention iCAN-DO compared to SC, on symptoms of anxiety and depression, and health-related quality of life (HRQoL) in individuals with cancer who report symptoms of anxiety and/or depression, shortly after diagnosis.

## 2 Method and material

### 2.1 Design

The AdultCan was a multicenter randomized clinical trial (Mattsson et al., 2013; Hauffman et al., 2017). The results for the primary endpoint (10 months) have been reported (Hauffman et al., 2020a). The present study concerns the evaluation of the 18- and 24-month follow-ups and is presented according to the CONSORT statement (Schulz et al., 2010). The AdultCan study was conducted in accordance with the Helsinki Declaration (World Medical Association, 2008), approved by the Swedish Ethical Review Authority (Dnr 2012/003), and registered in ClinicalTrials.gov (registration number: NCT-01620681).

### 2.2 Recruitment, screening, and randomization

**Fig. 1:**
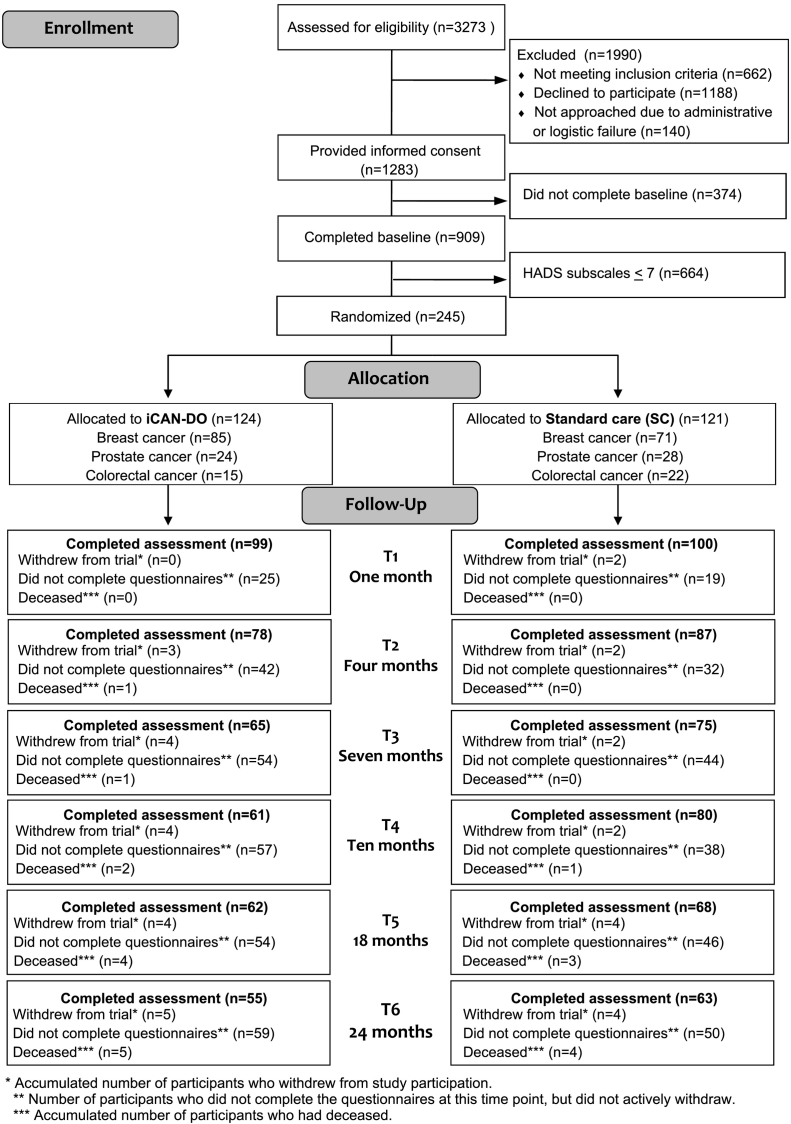
CONSORT diagram of enrollment, participants, and completed assessments in the U-CARE AdultCan trial.

Individuals were eligible if newly diagnosed (<6 months) with any stage of breast, colorectal, or prostate cancer, or with a relapse of an earlier curatively treated colorectal cancer, at four hospitals in mid-Sweden from 2013 to 2016. Exclusion criteria were cognitive impairment, inability to understand Swedish, expected survival < 3 months, Karnofsky performance status < 40, or severe depression or suicide ideation according to the Montgomery-Åsberg Depression Rating Scale (MADRS-S) (Fantino and Moore, 2009). Individuals with self-reported symptoms of anxiety and/or depression, i.e., >7 points on either of the Hospital Anxiety and Depression Scale (HADS) subscales (HADS-A and HADS-D) (Zigmond and Snaith, 1983), were randomly assigned in the U-CARE-portal to either iCAN-DO plus SC or only SC (Fig. 1) using a computer-generated permuted block method, stratified for curative or palliative treatment, and concealed to all research staff.

### 2.3 Sample size calculation

A minimal important difference in HADS-A or HADS-D corresponds to a 20 % difference/change (Puhan et al., 2008). This equals to about a two point change in the two HADS subscales respectively, according to our previous study (Thalén-Lindström et al., 2017). Sixty-five participants per group were needed to obtain 80 % power (alpha 0.05) to detect a 20 % mean score difference between groups after treatment. Since a large proportion of participants can be expected to drop out from internet-based studies (Eysenbach, 2005), we set out to include twice this number (Fig. 1).

### 2.4 The internet-based stepped care intervention iCAN-DO

iCAN-DO was developed in collaboration with staff within cancer care and individuals with a cancer diagnosis (Hauffman et al., 2017). iCAN-DO was delivered through the U-CARE-portal, and all contacts with participants occurred through written asynchronous communication. All participants randomized to iCAN-DO had access to Step 1 (psychoeducational content) from randomization and throughout the study period (24 months). Patients with persistent symptoms of anxiety or depression (>7 on any of the HADS subscales) at 1, 4, and/or 7 months after randomization were offered Step 2, a 10-week guided internet-based cognitive-behavioral therapy program (iCBT) in addition to Step 1. All participants randomized to iCAN-DO had access to SC.

#### 2.4.1 Step 1: psycho-education

Step 1 comprised a library, a peer support section, and an “Ask an Expert” feature. The library comprised 16 modules including information about cancer and cancer treatment, and psycho-educative material (in audio-visual and text format) about common problems surrounding cancer, such as anxiety, depression, fatigue, pain, and sleeping issues. The participants had access to all modules during the entire 24 months period.

The psycho-educative content aimed to describe how a symptom via a vicious cycle can affect thoughts, feelings, and/or behaviors in a way so that symptoms are maintained or even worsened, and how self-care strategies may break this cycle. Advice about when to seek professional treatment and information about treatment options were also provided. The peer-support section comprised a moderated discussion forum covering various themes. “Ask an Expert” meant that participants could pose questions to a nurse and/or read others' anonymized questions and answers in the FAQ (frequently asked questions).

#### 2.4.2 Step 2: iCBT

Participants who took part of the iCBT received a list of 15 modules covering common cancer-related problem such as depression, worry, rumination, etc., and could choose the problem area/s with which they wanted to work. The participant could work with all modules, but only two at a time. The treatment was highly structured and followed established CBT treatment manuals in each respective area (e.g., behavioral activation for depression and exposure therapy for anxiety); moreover, it included exercises, assignments, self-monitoring, and weekly asynchronous online contact with a psychologist. Participants could only participate in Step 2 (iCBT) once.

### 2.5 Standard care

SC included the regular activities routinely offered at the respective hospital, i.e., information about the disease, the treatment, possible side effects of the treatment, and what the individual can do to prevent and/or relieve symptoms and side effects. SC also included the opportunity, on an as-needed basis, to talk to a social counsellor or deacon/priest from the hospital church.

### 2.6 Measures

Data were collected at randomization and 1, 4, 7, 10, 18, and 24 months thereafter via the U-CARE-portal (for more detailed information, see Hauffman et al., 2020a). The present study mainly reports data collected at assessments 18 and 24 months after randomization. Well-known self-report questionnaires with good psychometric properties were used (see 2.6.2, 2.6.3).

#### 2.6.1 Medical and socio-demographic background data

Medical background data were obtained from national quality registers. Data on socio-demographics were self-reported with questions developed for the study.

#### 2.6.2 Primary outcomes: symptoms of anxiety and depression

The primary outcomes were assessed with HADS (Zigmond and Snaith, 1983), consisting of the two sub-scales: anxiety (HADS-A) and depression (HADS-D), at all assessment points. Both scores of the sub-scales and the HADS classification (≤7: non-case, 8–10: doubtful case, and >10: clinical case) were used.

#### 2.6.3 Secondary outcomes: health-related quality of life, cancer-related fatigue, and insomnia

HRQoL was assessed with EORTC QLQ-C30 (Aaronson et al., 1993), cancer-related fatigue by FACIT-F (Webster et al., 2003), and insomnia with ISI (Morin et al., 2011) at 18 and 24 months.

### 2.7 Statistical analysis

**Fig. 2:**
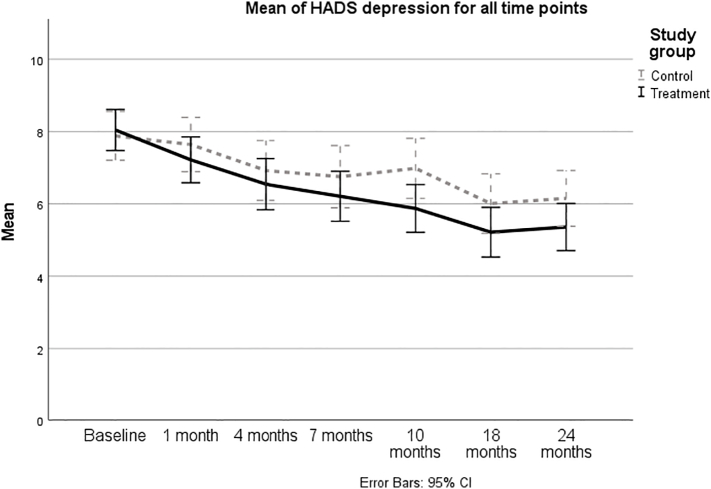
Symptoms of depression measured by HADS-D (mean values and 95 % confidence intervals) over the course of the study.

**Fig. 3:**
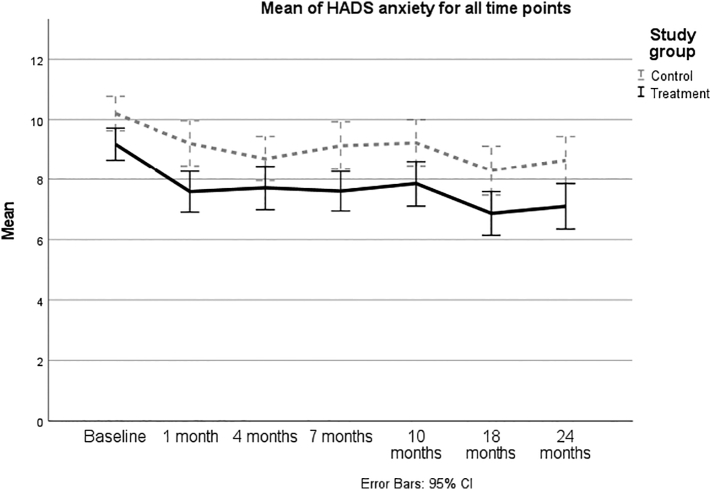
Symptoms of anxiety measured by HADS-A (mean values and 95 % confidence intervals) over the course of the study.

**Fig. 4:**
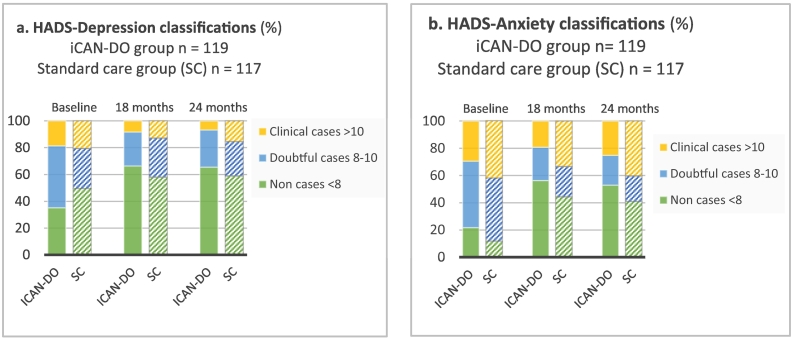
Changes over time according to the classification of HADS Depression (a) and HADS Anxiety (b). Individuals only had to be cases on one of the anxiety or depression subscales to be included in the study and therefore may be non-cases on one subscale at baseline. Abbreviation: HADS = Hospital anxiety and depression scale.

The analyses were performed in SPSS version 26 and R version 4.1.1. Total or composite scores for the outcomes were calculated according to published instructions. The missing value in a sub-scale comprising several items was imputed by the average of the remaining items of that sub-scale, if at least half of the items were valid (Fayers et al., 2001). Missing data were imputed using the last rank carried forward method (LRCF) (O'Brien et al., 2005), which allows for missing participants to keep their rank in relation to the others. Data collected with questionnaires completed at baseline, 18, and 24 months were analyzed with descriptive statistics. Data from all assessments (baseline, 1, 4, 7, 10, 18, and 24 months) were utilized in two linear mixed models, one model for depression and one for anxiety, with independent variables study group, time as a categorical variable and a random intercept for subject ID. A timepoint times study group interaction term was included as well to enable separate estimates of the effects at 18 and 24 months for the two main outcome measures respectively. All models were adjusted for baseline values, age, and gender. Effect sizes were calculated using Cohen's *d*, where a point measure of at least 0.20, 0.50, and 0.80 were considered a small, medium, and large effect size, respectively (Cohen, 1992). For HADS, clinically significant differences/changes were calculated using defined minimal clinically important differences (MCIDs) (Puhan et al., 2008). HADS meanscores (with 95 % confidence intervals) are presented for all assessments in Fig. 2, Fig. 3 to show development over time. Furthermore, differences in HADS classification (non-case vs. doubtful or clinical case) at different time points were also analyzed using Chi^2^. A visualization was also made for all three classifications (non-case, doubtful case, clinical case) (Fig. 4). All data were analyzed according to intention-to-treat (ITT). Due to a large number of missing data over time, sensitivity analyses were carried out for *complete cases*, i.e., those who completed all assessments, and *per protocol*, i.e., in the intervention group, only cases that adhered to the intervention were included in the analysis. Adherence was defined as:

(a) visiting at least three modules in the library, (b) visiting at least one module and one of the other functions (FAQ, Forum, Chat), or (c) completed at least two modules in iCBT.

## 3 Results

Of the 245 participants randomized, 118 completed the 24-month follow-up (Fig. 1), which implies an attrition rate of 51.8 % (iCAN-DO 55.6 %; SC 47.9 %; *p* = 0.23). There was no difference between the groups iCAN-DO and SC regarding socio-demographic or medical factors (Table 1). Seventy-three of the 124 participants (59 %) in iCAN-DO were deemed to have adhered to the intervention before the 10-month assessment. Descriptive data for baseline, 18 and 24 months, respectively, and effect sizes for between-group differences, are presented in Table 2, Table 3.

**Table 1:** Demographics and clinical characteristics for individuals randomized to iCAN-DO or standard care.

Characteristic	Randomized (n = 245)		Completed 24-month assessment (n = 117)	
	iCAN-DO (n = 124)	Standard care (n = 121)	iCAN-DO (n = 55)	Standard care (n = 63)
*Age, years*				
Mean (SD)	57.3 (10.6)	57.0 (10.7)	60.4 (9.3)	58 (10.9)
Min-max	29–86	33–75		

*Sex n (%)*^a^				
Female	93 (75)	81 (67)	39 (71)	37 (59)
Male	31 (25)	40 (33)	16 (29)	26 (41)

*Marital status n (%)*^a^				
Single/divorced/widowed	20 (16)	13 (11)	3 (5)	5 (8)
Married/cohabiting	95 (77)	98 (81)	48 (87)	52 (83)
Living apart together	6 (5)	6 (5)	3 (5)	3 (5)
Other	3 (2)	4 (2)	1 (2)	3 (5)

*Education n (%)*^a^				
Elementary school	18 (15)	23 (19)	8 (15)	12 (19)
High school	36 (29)	42 (35)	14 (25)	23 (37)
University/university college ≤ 3 years	29 (23)	28 (23)	10 (18)	14 (22)
University/university college > 3 years	41 (33)	28 (23)	23 (42)	14 (22)

*Country of birth n (%)*^a^				
Sweden	110 (89)	112 (93)	47 (85)	58 (92)
Outside Sweden	14 (11)	8 (7)	8 (15)	5 (8)

*Tumor origin, distant metastasis, and planned treatment n (%)*^b^				
Breast cancer	84 (68)	71 (59)	37 (67)	35 (56)
Distant metastasis	2 (2)	1 (1)		
Breast conserving surgery	55 (65)	47 (66)		
Mastectomy	27 (32)	23 (32)		
Chemotherapy	34 (41)	36 (51)		
Radiotherapy	78 (93)	63 (89)		
Endocrine treatment	60 (71)	54 (76)		
Antibody treatment	8 (10)	8 (10)		
Prostate cancer	24 (20)	28 (23)	14 (25)	20 (32)
Distant metastasis	1 (4)	3 (11)		
Prostatectomy	7 (32)	10 (36)		
Radiotherapy	12 (55)	10 (36)		
Endocrine treatment	8 (36)	8 (29)		
Colorectal cancer	14 (12)	22 (18)	4 (7)	8 (13)
Distant metastasis	2 (14)	2 (9)		
Segmental colon resection	13 (93)	19 (95)		
Stoma	4 (29)	9 (45)		
Chemotherapy	6 (43)	16 (80)		
Radiotherapy	5 (36)	12 (60)		

*Monthly income n (%)*				
Low < 1244 €	5 (4)	5 (4)	4 (7)	0 (0)
Average 1244–4976 €	82 (66)	79 (65)	30 (55)	38 (60)
High > 4976 €	5 (4)	3 (3)	3 (5)	1 (2)
Unknown (not responded)	32 (26)	34 (28)	18 (33)	24 (38)

*Psychosocial support n (%)*^c^				
Yes	7 (6)	7(6)	9 (16)	11 (17)
No	117(94)	114 (94)	46 (84)	52 (83)

*Currently using psychotropic drugs n (%)*				
Yes	24 (19)	24 (20)	14 (25)	14 (22)
No	100 (81)	97 (80)	41 (75)	49 (78)

*Computer experience n (%)*				
Very inexperienced	5 (4)	6 (5)	1 (2)	2 (3)
Quite inexperienced	12 (10)	12 (10)	6 (11)	8 (13)
Quite experienced	54 (44)	45 (38)	24 (44)	23 (36)
Very experienced	52 (42)	57 (48)	24 (44)	30 (48)

a Where numbers in a category do not add up to n or 100%, there is missing data.

b Individuals may have been planned for more than one treatment modality.

c Psychosocial support was defined as seeing a therapist at least once a month.

**Table 2:** Mean values and standard deviations for primary outcomes at baseline, 18, and 24 months. Change scores and adjacent between group effect sizes are also presented.

	Baseline		18 months					24 months				
	iCAN-DO (n = 120)	SC (n = 118)	iCAN-DO (n = 120)		SC (n = 118)		ES	iCAN-DO (n = 119)		SC (n = 117)		ES
	M (SD)	M (SD)	M (SD)	Δ BL (SD)	M (SD)	Δ BL (SD)	*d*	M (SD)	Δ BL (SD)	M (SD)	Δ BL (SD)	*d*
ITT^a^												
HADS Depression	8.0 (3.1)	7.9 (3.7)	5.2 (3.8)	−2.8 (3.5)	6.1 (4.6)	−1.8 (3.9)	−0.29⁎	5.4 (3.6)	−2.7 (3.5)	6.2 (4.2)	−1.7 (3.6)	−0.27
HADS Anxiety	9.1 (3.0)	10.2 (3.1)	6.8 (4.0)	−2.3 (3.4)	8.3 (4.5)	−1.9 (3.7)	−0.13	7.1 (4.2)	−2.1 (3.5)	8.6 (4.3)	−1.6 (3.8)	−0.14
Complete cases^b^	n = 62	n = 68	n = 62		n = 68			n = 55		n = 63		
HADS Depression	7.5 (3.1)	8.1 (3.9)	5.2 (3.7)	−2.3 (3.9)	6.4 (4.2)	−1.7 (3.6)	−0.18	5.6 (3.2)	−2.0 (4.0)	7.3 (3.8)	−0.9 (3.8)	−0.27
HADS Anxiety	9.0 (2.6)	9.6 (3.2)	7.0 (3.5)	−2.0 (3.4)	8.6 (4.1)	−1.0 (3.6)	−0.28⁎	7.5 (3.7)	−1.3 (3.4)	9.5 (3.8)	−0.3 (3.7)	−0.27⁎
Per-protocol^c^	n = 78	n = 118	n = 78		n = 118			n = 77		n = 117		
HADS Depression	7.8 (3.2)	7.9 (3.7)	5.4 (3.8)	−2.4 (3.7)	6.1 (4.6)	−1.8 (3.9)	−0.16	5.6 (3.5)	−2.3 (3.6)	6.2 (4.2)	−1.7 (3.6)	−0.14
HADS Anxiety	9 (2.7)	10.2 (3.1)	7.2 (3.9)	−1.9 (3.4)	8.3 (4.5)	−1.9 (3.7)	−0.01	7.5 (4.1)	−1.6 (3.6)	8.7 (4.3)	−1.6 (3.8)	−0.01

NOTE: A high score represents a higher level of symptoms/problems in all scales. Between group effect sizes (ES) are calculated from baseline to follow-up. A negative ES indicates a lowering of symptoms or function (depending on the measure), and a positive ES indicates an increase.

Abbreviations: HADS = Hospital Anxiety and Depression Scale; ITT = intention-to-treat; BL = baseline; M = mean; SD = standard deviation; CI = confidence intervals.

a ITT = intention to treat, including all participants who completed baseline. Data were imputed using last rank carried forward method.

b Complete cases = Individuals who completed the assessment and had no missing values.

c Per-protocol = In iCAN-DO only participants adherent to the intervention.

⁎ Statistically significant intervention effects in the linear mixed model analyses.

**Table 3:** Mean values and standard deviations for all secondary outcomes at baseline, 18, and 24 months. Change scores and adjacent between group effect sizes are also presented.

ITT^a^	Baseline		18 months					24 months				
	iCAN-DO (n = 120)	SC (n = 118)	iCAN-DO (n = 120)		SC (n = 118)		ES	iCAN-DO (n = 119)		SC (n = 117)		ES
	M (SD)	M (SD)	M (SD)	Δ BL (SD)	M (SD)	Δ BL (SD)	*d*	M (SD)	Δ BL (SD)	M (SD)	Δ BL (SD)	*d*
EORTC QLQ C30												
Global health	48.5 (21.4)	47.5 (22.5)	68.2 (21.5)	19.7 (23.2)	64.7 (26.7)	17.1 (23.8)	0.11	67.1 (21.1)	18.5 (23)	62.6 (24.2)	14.9 (24.9)	0.15
Physical functioning	77.4 (20.7)	80 (17.5)	84.7 (18.9)	7.3 (16.1)	84.5 (18.3)	4.5 (15.5)	0.18	82.9 (19.1)	5.2 (18.1)	83.9 (18.1)	3.9 (17)	0.08
Role functioning	52.8 (32.4)	59.2 (33.9)	83.9 (25.5)	31 (32.1)	83.8 (25.1)	24.6 (36.6)	0.19	84.3 (23.2)	31.6 (32.2)	81.1 (28.3)	21.4 (41.9)	0.27
Emotional functioning	60.6 (18.2)	56.1 (21.4)	73.7 (22.6)	12.8 (20.9)	65.4 (24.1)	9.4 (20.4)	0.17	72.7 (25.2)	11.9 (23.2)	65.4 (25.8)	9.2 (22.4)	0.12
Cognitive functioning	65.7 (24.7)	64.7 (28.1)	78.7 (20.4)	15.1 (24.5)	75.4 (25.2)	11.7 (25.3)	0.13	79.8 (18.7)	16.1 (22.4)	75.1 (23.5)	11.3 (26.5)	0.19
Social functioning	58.1 (29.1)	60.3 (28.4)	84 (18.1)	26 (30.1)	76 (28.1)	15.7 (29.1)	0.35⁎	84.6 (17.6)	26.7 (30.5)	73.5 (29.5)	13 (32.4)	0.44⁎
Appetite loss	35 (31)	19.6 (27.1)	6.1 (14.2)	−18.9 (32.3)	11 (20.4)	−9.1 (25.4)	−0.34	8.1 (17.8)	−16.8 (34.4)	10.2 (18.8)	−9.5 (26.3)	−0.24
Constipation	14.1 (25.4)	17.6 (26.5)	9.4 (20.4)	−4.7 (24.2)	12.4 (24.6)	−5.1 (30.2)	−0.01	10.6 (21.7)	−3.6 (28.7)	11.1 (24)	−6 (29.4)	−0.08
Diarrhea	15.8 (27)	17 (24.2)	6.4 (18)	−9.4 (29.3)	10 (23.3)	−7.1 (27)	−0.08	7.8 (18.2)	−8.1 (29.7)	7.4 (19.7)	−9.7 (24.8)	−0.06
Dyspnea	34.6 (30.4)	26.7 (27.7)	17.1 (25.2)	−17.5 (33.2)	18.8 (26.7)	−7.9 (32.9)	−0.29	17.6 (25.9)	−16.5 (33.3)	21 (27.5)	−5.4 (33.1)	−0.34
Fatigue	51.5 (26)	48.1 (25.2)	28.5 (25.3)	−23 (26.1)	30.9 (24.9)	−17.3 (24.9)	−0.22	26.4 (25.4)	−24.9 (28.6)	29.4 (25.1)	−18.3 (25.8)	−0.24
Nausea and vomiting	13.2 (18.1)	10.8 (13.9)	2.8 (8.2)	−10.4 (19.3)	4.1 (10.5)	−6.7 (15)	−0.22	2.6 (8.1)	−10.8 (19.1)	4 (11.2)	−6.7 (15.4)	−0.23
Pain	33.8 (28.4)	30.1 (28.4)	18.1 (26)	−15.7 (32.3)	17 (23.6)	−13.1 (27.8)	−0.09	19.4 (25.2)	−14.3 (30.5)	18.7 (24.6)	−11 (29.2)	−0.11
Sleep problems	42.2 (31.5)	45.4 (32.3)	26 (24.9)	−16.2 (33.3)	34.4 (28.3)	−11.1 (31.9)	−0.16	15.9 (27.4)	−26 (38)	27.9 (32.2)	−17.1 (38.1)	−0.24⁎
Financial difficulties	24.4 (33.4)	20.9 (29.7)	22.2 (34.4)	−2.2 (26.6)	15.6 (25.3)	−5.5 (26)	−0.12	26.1 (39.1)	1.5 (25.8)	21 (34.8)	−0.5 (32.2)	−0.07
ISI (insomnia)	11.5 (6)	12.4 (5.9)	9.6 (7.1)	−1.9 (5.9)	10.9 (7.8)	−1.5 (5.8)	−0.06	9.1 (6.3)	−2.3 (5.5)	10.6 (7.2)	−1.8 (5.2)	−0.10
FACIT-F (fatigue)	30.8 (10.4)	30.9 (11.3)	36.9 (10.8)	6.1 (10.4)	36 (12.1)	5.1 (10.6)	0.09	35.8 (11.5)	5 (10.9)	35.7 (11.6)	4.7 (10.4)	0.03

NOTE: A high score represents a higher level of symptoms/problems in all scales, except from the function sub-scales in the EORTC QLQC30 and FACIT-F. Between group effect sizes (ES) are calculated from baseline to follow-up. A negative ES indicates a lowering of symptoms or function (depending on the measure), and a positive ES indicates an increase.

Abbreviations: BL = baseline; M = mean; SD = standard deviation; EORTC QLQ C30 = European Organization for the Research and Treatment of Cancer Quality of Life Questionnaire; ISI = Insomnia Severity Index; FACIT-F = The Functional Assessment of Chronic Illness Therapy – Fatigue.

a ITT = Intention to treat, including all participants who completed baseline. Data were imputed using last rank carried forward method.

⁎ Statistically significant intervention effects in the linear mixed model analyses.

### 3.1 Symptoms of anxiety and depression

There was a statistically significant effect of iCAN-DO on symptoms of depression at 18 months (*p* = 0.049; Table 4). The average within group decreases from baseline in symptoms of depression were 2.8 and 1.8, respectively, in the two groups at 18 months, and 2.7 and 1.7, respectively, at 24 months. Thus, the iCAN-DO group, but not the SC group, decreased their scores, on average, at a clinically significant level (>2). The difference between groups implied a small effect size at both time points (both *d*s < 0.30; Table 2; Fig. 2).

**Table 4:** Results from linear mixed models for symptoms of depression and anxiety, health-related quality of life, insomnia, and fatigue at 18 months and 24 months, respectively. All models are adjusted for baseline values, age and gender. A timepoint times treatment group interaction was included in the models. Each model includes a random intercept for subjects.

	18 months				24 months			
	Diff	CI −95 %	CI +95 %	*p*	Diff	CI −95 %	CI +95 %	*p*
HADS-Depression	−0.902	−1.800	−0.003	**0.049**	−0.799	−1.697	0.100	0.081
HADS-Anxiety	−0.761	−1.677	0.156	0.104	−0.804	−1.721	0.113	0.086
EORTC QLQ C30								
Global health	2.691	−2.604	7.985	0.319	3.525	−1.770	8.820	0.191
Physical functioning	1.680	−2.162	5.522	0.391	0.589	−3.253	4.431	0.764
Role functioning	2.276	−4.620	9.173	0.517	5.205	−1.691	12.102	0.139
Emotional functioning	4.885	−0.472	10.242	0.074	3.960	−1.397	9.317	0.147
Cognitive functioning	2.766	−2.686	8.217	0.320	4.127	−1.325	9.578	0.138
Social functioning	7.587	1.303	13.872	**0.018**	10.467	4.182	16.751	**0.001**
Appetite loss	−4.988	−10.414	0.437	0.071	−2.303	−7.728	3.123	0.405
Constipation	−1.742	−7.828	4.343	0.574	0.679	−5.407	6.764	0.827
Diarrhea	−2.545	−8.154	3.065	0.374	1.260	−4.350	6.869	0.659
Dyspnea	−4.375	−11.166	2.416	0.206	−6.062	−12.853	0.728	0.080
Fatigue	−3.714	−9.199	1.772	0.184	−4.411	−9.896	1.074	0.115
Nausea and vomiting	−1.748	−4.935	1.438	0.282	−1.753	−4.940	1.434	0.281
Pain	−0.761	−7.070	5.549	0.813	−1.188	−7.497	5.122	0.712
Sleep problems	−6.949	−14.277	0.379	0.063	−10.041	−17.369	−2.713	**0.007**
Financial difficulties	3.119	−2.850	9.088	0.305	1.843	−4.126	7.813	0.544
Insomnia Severity Index (ISI)	−0.517	−1.890	0.856	0.460	−0.695	−2.068	0.678	0.320
FACIT-F	0.671	−1.692	3.035	0.577	0.157	−2.206	2.521	0.896
Complete cases								
HADS-Depression	−1.028	−2.153	0.097	0.073	−1.113	−2.269	0.042	0.059
HADS-Anxiety	−1.438	−2.550	−0.327	**0.011**	−1.649	−2.794	−0.503	**0.005**
Per-protocol								
HADS-Depression	−0.911	−1.914	0.093	0.075	−0.703	−1.706	0.300	0.169
HADS-Anxiety	−0.668	−1.689	0.353	0.199	−0.627	−1.648	0.394	0.228

Values in bold denote statistical significance at the p < 0.05 level.

The effect of iCAN-DO on symptoms of anxiety was not significant, either at 18 or 24 months (*p* = 0.10 and *p* = 0.09; Table 4). The average within group decreases from baseline in symptoms of anxiety were 2.3 and 1.9, respectively, in the two groups at 18 months, and 2.1 and 1.6, respectively, at 24 months. Thus, the iCAN-DO group, but not the SC group, decreased their scores, on average, at a clinically significant level (>2). The difference between groups implied a very small effect size at both time points (both *d*s < 0.15; Table 2; Fig. 3).

At baseline, a larger proportion of participants in iCAN-DO reported symptoms of depression to, at least, a doubtful (mild) level (65 %) compared to SC (50 %; *p* = 0.026). The proportion who reported no symptoms increased from 35 % to 66 % at 18 and 24 months, respectively, in the iCAN-DO group, and from 50 % to 58 % and 59 % at 18 and 24 months, respectively, in the SC group (Fig. 4).

Regarding symptoms of anxiety, a smaller proportion of participants in the iCAN-DO group reported symptoms at, at least, a doubtful level (78 %) at baseline compared to SC (88 %; *p* = 0.043). The proportion who reported no symptoms increased from 22 % to 56 % and 53 % at 18 and 24 months, respectively, in the iCAN-DO group and from 12 % to 44 % and 41 % at 18 and 24 months, respectively, in the SC group (Fig. 4).

#### 3.1.1 Sensitivity analyses

The effect of the intervention on symptoms of depression was not confirmed in the complete case-analysis, either at 18 months (*p* = 0.07) or 24 months (*p* = 0.06). However, an intervention effect was seen for symptoms of anxiety at both 18 (*p* = 0.01) and 24 months (*p* = 0.005). Per-protocol analyses did not result in any intervention effects.

### 3.2 HRQoL, fatigue, and insomnia

There were significant effects of iCAN-DO on two HRQoL aspects; on social functioning at both 18 (*p* = 0.02) and 24 months (*p* = 0.001), and on sleep problems at 24 months (*p* = 0.01). Insomnia and fatigue decreased in both groups, but there was no between-group difference, either at 18 or 24 months.

## 4 Discussion

### 4.1 Results

The iCAN-DO intervention has previously been shown to have short-term (10 months) effects on symptoms of depression but not anxiety (Hauffman et al., 2020a). In the present long-term follow-up, the symptoms of depression again were lower in the iCAN-DO group compared with the SC group at both follow-ups, corresponding to a clinically relevant change (>2p) and a small intervention effect (*d* = 0.27–0.29). The linear mixed model including all measurement points revealed a statistically significant intervention effect at 18 but not at 24 months after randomization. A complete case-analysis did not confirm this result but revealed an effect on anxiety at both time points. Per-protocol analyses only including participants who adhered to the intervention showed no significant results at any of the follow-ups.

To our knowledge, the present RCT is the very first to report effects of an internet-based stepped care intervention at a 24-month follow-up. According to Wang et al. (2020), the hitherto longest follow-up period of psycho-educative interventions has been 12-months and only in one study. The reason that the intervention gave better effects on depression than anxiety is not clear, but one hypothesis is that Step 1 included behavioral activation components (e.g., social and physical activities and daily planning), which may have had effect on depression rather than anxiety symptoms. This result is also congruent with the results of the systematic review of internet-based psycho-educational interventions reported by Wang et al. (2020) that concluded intervention effects on symptoms of depression but not on distress.

Another explanation might be that the mechanisms behind anxiety are related to the real threat of the illness i.e. fear of cancer recurrence (FCR), defined as a fear, worry or concern that cancer may relapse or progress (Lebel et al., 2016). Step 1 did include anxiety self-management modules, such as worry time, and relaxation, but they did not target FCR specifically. Furthermore, some individuals cope with their cancer by avoiding medical and psychosocial information (Lupton, 2013). For example, Cillessen et al. (2020) found that non-users of an online mindfulness intervention had higher FCR at baseline than users. Because attrition and non-usage was very high in the present study, FCR might explain the lack of findings with respect to anxiety. Further, it has been previously shown that symptoms of anxiety change over time and could even increase in the post-treatment period (Lopes et al., 2022; Watts et al., 2014). FCR on the other hand, does not appear to decrease over time (Simard et al., 2013). Thus, the iCAN-DO material could potentially be improved by adding content to target FCR specifically.

Yet another explanation could be the use of HADS to measure anxiety. HADS was originally created as a screening instrument for symptoms of anxiety and depression in patients with a somatic condition, e.g. cancer, and is now one of the most used instruments worldwide in this capacity. It has received some critique regarding its sensitivity to change and its factor structure (e.g. Coyne and van Sonderen, 2012). However, the critique is not uniform and HADS has performed especially well in studies with somatically ill patients (Bjelland et al., 2002; Doyle et al., 2012; Annunziata et al., 2020).

The iCAN-DO intervention also showed some effects on the HRQoL aspects social functioning and sleep problems. This is in line with a literature review (Bouma et al., 2015) that found some effects on quality of life in individuals with cancer who had worked with an internet-based support program. However, the reviewed studies used different instruments, which debilitates comparisons. Nevertheless, it seems logical that a decrease in symptoms of depression or anxiety could be accompanied by improved aspects of HRQoL.

To receive support through an internet-based application has both advantages and disadvantages vis-à-vis in-person delivered support. The qualitative evaluations from the AdultCan study showed that the participants considered iCAN-DO being reliable and trustworthy and an important complement to SC, but they also expressed that it should be more personalized (Hauffman et al., 2020b). Igelström et al. (2020) found that the perceived benefits related to the simple system, multiple delivery modes, and the high accessibility. On the other hand, the initial log-in procedure with double authentication and technical problems (e.g., being logged out and plug-in struggles) decreased the motivation for use and may be an explanation for the high attrition rate. This has also been highlighted in a narrative synthesis of reviews on internet-based psychological interventions for cancer survivors (Leslie et al., 2022) where barriers to study participation and intervention adherence were reported to reflect e.g. misgivings concerning technology and a low tailoring of the intervention. Rather, superior efficacy was seen in multicomponent and tailored interventions, and interventions that had greater communication with a health care professional.

A considerable strength of the present study is that only individuals with symptoms of anxiety and/or depression were included. Individuals without elevated symptoms of depression or anxiety could hardly decrease their symptom levels further, no matter how effective the intervention. This is an example of the floor-effect seen in previous studies (Sanjida et al., 2018). It is also important that resources are invested where they have the greatest benefit, which is likely not in asymptomatic patients. Another strength is the psycho-educational content with a stepped care approach; however, only four of the 76 individuals that were offered iCBT participated in at least one session. The use of iCBT as a sole intervention for depression and anxiety in cancer patients has been evaluated in a meta-analysis (Liu et al., 2022). Pooled data from 13 studies with intervention lengths of 2–24 weeks showed that iCBT lasting 12 weeks or shorter, particularly when therapist-guided, and with ≥5 modules, relieved depressive and anxiety symptoms in cancer patients. In previous interviews, participants in iCAN-DO have expressed sufficient support from relatives and friends as one reason for turning down the offer of iCBT provided in Step 2. They also described symptoms and needs as dynamic and changing during the cancer trajectory, and that the division into modules in the iCBT was difficult, as all symptoms interact (Hauffman et al., 2020b). Thus, due to the high rejection rate of iCBT, the intervention comprised only Step 1 for most participants. Step 1, in the iCAN-DO intervention, also demands fewer resources as there is no therapist-guidance. Our results are mainly due to Step 1, which may therefore be considered for implementation in clinical practice without Step 2, thus avoiding large expenses. This may provide individuals with support at their time of choice and regardless of how far from the clinic they live. However, since study participants using the U-CARE portal have expressed varying preferences (written messages, face-to-face) when it came to communication with health care (e.g. the psychologist) (Hauffman et al., 2020b; Wallin et al., 2016), it is recommended that patients are offered either face-to-face CBT or iCBT, when internet-based psycho-educative material is not enough to reduce the symptoms of depression and anxiety.

### 4.2 Study limitations

The drop-out rates in internet-based interventions for individuals with cancer, similar to this one, have varied (Wang et al., 2020). Even though many interventions have had shorter follow-up times than in the present study, close to, and above, 50 % drop-out rates have been reported (Wang et al., 2020). A follow-up 24 months after randomization is the hitherto longest follow-up period after an internet-based intervention with stepped-care and a drop-out rate of 52 % is therefore difficult to evaluate due to lack of comparisons. There is also a potential risk of bias since the missingness may not be completely at random. However, the attrition rates did not differ between the iCAN-DO and the SC group (*p* = 0.23) from baseline to the last follow-up. Nonetheless, the ITT analysis approach is therefore important. The choice to use last rank carried forward imputation was based on recommendations by O'Brien et al. (2005), as it was considered to give the most accurate representation of missing data by allowing missing participants to keep their rank in relation to the others. Mixed models assume missing at random and thereby that missing participants have a similar development as remaining participants. This may be problematic especially when data are missing due to death. The attrition rate over time did not differ between iCAN-DO and standard care, and the participant's total HADS score did not, at any previous time point, predict drop-out rate at 24 months. However, those who dropped-out from the study were younger, more likely single, and more often diagnosed with colorectal cancer. Thus, we preferred LRCF since it is considered less sensitive to non-missing at random (NMAR), and as it is regarded as a conservative estimate.

In addition to ITT analyses, sensitivity analyses using complete cases only and those adhering to the iCAN-DO, as per-protocol, were performed (Bennett, 2001). The complete cases analyses did not confirm the effect on symptoms of depression but an effect was evident for symptoms of anxiety. There is a possibility that this reflects reduced statistical power due to small sample size (Andrade, 2022). The lack of effect in the per-protocol analyses indicates that improvement was not clearly related to the intervention activity. However, as the activity in the portal was relatively low with only 59 % of the participants adhering to the intervention, the per-protocol analysis also was likely under-powered.

Those who declined study participation were more likely to be older and having colorectal or advanced cancer. Hence, the findings are more generalizable to younger individuals with primarily breast and prostate cancer.

### 4.3 Conclusions

A stepped-care internet-based intervention that has previously shown positive short-term results for symptoms of depression also showed a positive long-term effect for symptoms of depression. The internet may provide an effective format of support to reduce symptoms of depression after cancer at the patients' own choice of time, regardless of the distance to a psycho-oncology clinic.

## Declaration of competing interest

The authors declare that they have no known competing financial interests or personal relationships that could have appeared to influence the work reported in this paper.
